# From Our Correspondents

**Published:** 1984-07

**Authors:** 


### From Our Correspondents

### The Jenner Lecture

The Jenner Lecturer this year was Sir Peter Medawar. In the title of his lecture he posed the question 'Is inoculation against cancer theoretically possible?' and he started immediately by expressing the opinon that he thought it should be.

He presented this complex subject with great lucidity, beginning with a graph showing the relationship between the incidence of breast cancer and the age of first pregnancy and showing that as the age advances so does the incidence of breast cancer. He explained it as follows—malignant cells contain active substances of genetic origin which are antigenic and are also present in normal embryo cells. These substances become dormant as the embryonic cells mature. When a tissue becomes malignant these substances are reawakened. The antibodies produced in the pregnant mother by embryonic antigen are capable of protecting against cancer but the timing of the antigen challenge is critical in relation to cancer.

Experiments have shown in animals that if a cancer has already developed then the immunological response can actually activate it. Mice subject to Methyl Cholanthrene induced tumours were divided into 3 groups. In the first group embryonic cells were injected before the methyl cholanthrene, in the second both were given together and in the third the cells were given after the methyl cholanthrene. Only in those mice given embryonic cells before the methyl cholanthrene was there a considerable level of protection. This experiment shows that immunological treatment can only be effective as a prophylactic measure and explains why immuno-therapy of established cancer has been unsuccessful. Most other tissues have been tested for their immunological activity against cancer but only cells taken from the thymus and from the testicle have been shown to be active. Mice brought up in a sterile environment have a higher incidence of spontaneous cancers than those exposed to the normally occurring bacteria present in a natural environment.

The problem facing researchers using the human animal is the very long life span. In mice for example results can be achieved in 2–3 years, in man conclusions can only be reached after 70–80 years. It will be many years before an immunological treatment for cancer is found.

Delivering such a lecture under his present disability was clearly tiring for Sir Peter and he did not feel able to prolong the effort by answering questions. If he had done so his large and fascinated audience could have kept him the whole evening trying to draw him out on the implications of what to most of us was a completely novel concept. We are very grateful to Sir Peter for giving us so much to think about and for the effort it must have cost him.

D. C. Bodenham

### The Future for Frenchay

Plans are afoot at Frenchay, which, if implemented, will change the face of the hospital over the next few years and beyond. The D.H.S.S. has approved the Phase I development of the re-building programme, and has allocated 5 million pounds towards the capital cost. Further phases are planned for the years to follow, but as yet these are unspecified. Phase I consists of a ward block of 258 beds to be built on the green sward outside the front of Ward 30, the Burns Unit, housing at present mostly children. Several of the existing wards will be emptied into the new block when completed, and then the old buildings will be demolished to await the development plans for Phase II.

The concept of a ward block originated five or six years ago, and plans have been slowly maturing on its structure and contents ever since. A model is being prepared for public display within the next few weeks to illustrate how the development will appear. It is hoped that it will generate considerable interest. A natural result already is that several opinions are circulating that it is not just the wards that need replacing, but that many other features at Frenchay Hospital need to be upgraded, and that the existing plans could stimulate a fresh look and further thought. The whole concept of hospital buildings is presently undergoing a change and a flexible approach may be needed. Time again will tell.

The second item is the proposed demolition of the stable block for which an application has already been lodged with the local district council. For those interested in local history, this building is part of the original house curtilage built about 200 years ago in the fine parkland, which now constitutes the grounds of Frenchay Hospital. The stable block in fact is of interest in that it has a high arched entrance to the inner courtyard for the passage of a coach and four, a style almost unique in the area. There are also some interesting round windows. The building is in local pennant stone, quarried in the neighbourhood, and having an attractive reddish-grey colour in it. Unfortunately the building has been neglected, and although it still serves a useful function in housing the farming and gardening equipment, the Authority regard it as beyond repair. However both the House, now District Headquarters, and the stable block, are

Listed Buildings Grade II. It may be that anyone interested in fine old listed buildings should look at that small part of our inheritance while the opportunity still presents. It may not be there in a few weeks time.

J.L.G.T.

**What Pathology Tests are Really Necessary?**

An unexpected meningitis found recently in our post-mortem room set me to wonder if laboratories spend their budget on the most effective pathology tests. Possibly visual inspection of the urine has historical precedence over post-mortem examinations. Certainly both antedate cytology, chromosome analysis, multichannel chemical pathology and even a humble haemoglobin. The patient in question had had a series of repeated and expensive investigations, and was eventually thought to have cerebral atrophy. An evidently increasingly unpopular[1] post-mortem examination was necessary to reveal the truth.

Not so long ago *The Lancet*[2] carried a Leading Article entitled 'Pathology Tests—too much of a good thing?' Paradoxically there is a constant rise of between 6 and 17% in the annual rate of requests for haematology, chemical and microbiology tests. And yet, countrywide, there is a continual decline in non-Coroner's Post-Mortem Examinations. So bad is this state of affairs that one is pained to see the limited experience of histopathology trainees when they write up their curricula vitae in search of the next post.

Why should this be? The concept of clinical review and audit is especially fashionable now. Surely even sophisticated biochemistry and the impressive non-invasive body images of today themselves require some check. Why not, if perchance the worst occurs, in the post-mortem room? There are pathologists available for such things.

Is it that the task of asking relatives for the ultimate pathological test is too taxing? If any examination was really necessary, surely this one cannot be neglected. Even colleagues with the chromatograms, monoclonal antibodies, antibiotic discs, and Coulter counters would agree about the importance of this last investigation. Maybe both hospital clinicians and general practitioners ought to make different use of their laboratory based colleagues. We are perfectly willing to oblige them, and even to arrange the subsequent Clinico-Pathological Conferences.

J.D.D.

**REFERENCES**

1. The general decline in rates of post-mortem examination is well documented in the past four decades in the U.K. The discrepancy between diagnosis in life and *post-mortem* has been the subject of several recent reports.
2. *The Lancet.* (1984) Leader. *i*, 1278.

**Holistic Medicine**

Isn't it fascinating how old terms keep recurring? Hippocrates was the first to suggest that man should be looked at as a whole and somehow over the course of centuries this concept has been lost—only to be regained at the end of the twentieth century!

We all know that much has been gained by our spell of scientific enquiry and by the understandable need to simplify problems by dividing them into self-contained small units; but much has been lost. We now have specialists who are expert in the diseases of parts of the body, a liver specialist, a cardiologist and so on. Even specialties like paediatrics are subdividing into paediatric neurology and paediatric endocrinology. Is all this gain?

Solzhenitsyn in Cancer Ward says 'But the patient's organism isn't aware that our knowledge is divided into separate branches. You see the organism isn't divided'. He goes on to suggest that the doctor ought to be an allrounder only to be met by Dontsova's despair at the seemingly impossible task of amassing the sum of knowledge gained by the specialists.

Perhaps specialism has gone too far for most patients.

It is therefore perhaps not surprising that holistic medicine has reappeared. The suggested rationale behind its reappearance is the need to look at patients needs in a more global way rather than the reductionist approach of the specialist. How much are the patients fears contributing to his illness? How much influence is his mother in law having on his reaction to his symptoms and so on. Man or his parts cannot be viewed in isolation. One other 'holistic' concept is that for most illnesses only part of the organism is 'unwell'; perhaps we, as doctors, should be concentrating at least as much on the remaining (larger) part of the organism that is healthy. Ought we to be encouraging this larger part of the patient towards better health and therefore aiding the rest of the body in its attempt at homoeostasis.

Is all this nonsense or are we painfully being encouraged to reassess and overcome our prejudices and our traditional teaching? General practice has traditionally approached patients in a holistic way. But even general practitioners have much to learn from the new discipline of holistic medicine; we need to broaden our concepts and to consider embracing such techniques as relaxation, meditation, diet and even visual imaging to help our patients manage their illhealth.

M.J.W.

### Cross Examination of an Expert Medical Witness

'Now, doctor, I would like to turn to your report. At the bottom of page six you say, "This situation was as far from normal as black is from white." By this situation I presume you mean the unfortunate predicament in which the plaintiff finds herself. You do? Good.

'I would now like to ask you a simple question, doctor. What do you mean by black? . . . Oh, I see, so black is a colour. How do you define blackness, doctor? For example, is there only one black or are there several shades of black? . . . I see, you tell me that there are several shades of black, of which some are less black than others. How would you best describe those shades of black which are lighter than the others? . . . So you would describe them as greyish, would you, doctor? Yes, you would. Thank you, doctor. Now let me turn to something else . . .'

(5 minutes later)

'A few moments ago, doctor, you told me that black was grey, did you not? You did not—I see. What you said was that some degrees of blackness were lighter than others. Did you use the word "greyish", doctor? You did. Good.

'Now let me turn to another question. Tell me, doctor, what do you mean in your report by "white"? I see, white too is a colour. We are having a colourful morning, aren't we?

'How do you define white, doctor? I realise it is difficult, but you are an expert, after all, are you not, doctor? Perhaps I can clarify it for you a little. Is there only one white, or are there several shades of white. I see, there are several shades of white.

'Tell me, doctor, what do you call the shades of white which are darker than the others. I see, you might call them greyish. So, therefore, black can be greyish, can it not; and white can be greyish, can it not? I do not wish to put words in your mouth, doctor. I am only trying to help my Lord to understand an important part of your report. You agree with me that in your evidence you have said that black can be greyish? You have also said that white can be greyish. You agree? Good. It must therefore follow, doctor, that black and white need not be all that different, if they can both be greyish, as you have given evidence, doctor. Would you agree? You do. Thank you. Thus there might even be occasions when they are the same, doctor—I'm only going by your evidence, doctor. You agree?

'Therefore, doctor, might it be possible for us to rewrite that sentence in your report as follows: "This situation was as near normal as black (or grey) is from white (or grey)." We can? Thank you, doctor. I have no further questions, my Lord.'

